# Single-cell landscape of peripheral immune cells in MASLD/MASH

**DOI:** 10.1097/HC9.0000000000000643

**Published:** 2025-04-21

**Authors:** Agnes Anna Steixner-Kumar, Diana Santacruz, Tobias Geiger, Werner Rust, Dennis Böttner, Oliver Krenkel, Ehsan Bahrami, George Okafo, Thomas F.E. Barth, Mark Haenle, Wolfgang Kratzer, Patrycja Schlingeloff, Julian Schmidberger, Heike Neubauer, Alec Dick, Markus Werner, Eric Simon

**Affiliations:** 1 Department of Global Computational Biology and Digital Sciences, Boehringer Ingelheim Pharma GmbH & Co.KG, Biberach, Germany; 2 Department of Cardiometabolic Research, Boehringer Ingelheim Pharma GmbH & Co.KG, Biberach, Germany; 3 Institute of Pathology, Ulm, Ulm University, Germany; 4 Department of Internal Medicine I, University Hospital Ulm, Ulm, Germany

**Keywords:** MASLD, MASH, liver, blood, scRNA-Seq, immune cells, B cells, neutrophils, myeloid-derived suppressor cells, immature immune cells

## Abstract

**Background:**

Metabolic dysfunction–associated steatotic liver disease (MASLD) progresses to metabolic dysfunction–associated steatohepatitis (MASH) and is a major cause of liver cirrhosis. Although liver inflammation is the hallmark feature of MASH versus MASLD, the involvement of the peripheral immune cell compartments in disease progression is poorly understood, and single-cell profiles of peripheral immune cells in MASLD/MASH are not known.

**Methods:**

Patients with MASLD/MASH and healthy volunteers have been prospectively enrolled in a cross-sectional study. Patients have been histologically stratified and further characterized by liver bulk RNA sequencing (RNA-Seq). Peripheral immune cells from patients and control blood samples have been comprehensively profiled using bulk and single RNA-Seq.

**Results:**

Twenty-two patients with fibrosis stage less than F3 have been histologically stratified into patients with low, medium, and high disease activity scores (NAFLD activity score [NAS]). In contrast to fibrosis, the NAS group correlated with noninvasive imaging readouts and blood biomarkers of liver damage and inflammation (ALT, AST). The prevalence of type 2 diabetes and obesity increased with the NAS stage. Bulk RNA-seq profiling of patient liver biopsies revealed gene signatures that were positively and negatively associated with NAS. Known marker genes for liver fibrosis where upregulated on RNA level. Blood bulk RNA-seq showed only moderate differences in patients versus healthy controls. In contrast, single-cell analysis of white blood cells revealed multiple alterations of immune (sub-)populations, including an increased abundance of immature B cells and myeloid suppressor cells in patients with MASLD/MASH as compared to healthy controls.

**Conclusions:**

The study gives new insights into the pathophysiology of MASLD/MASH already manifesting relatively early in peripheral immune cell compartments. This opens new avenues for the development of new biomarker diagnostics and disease therapies.

## Graphical abstract

**Figure:**
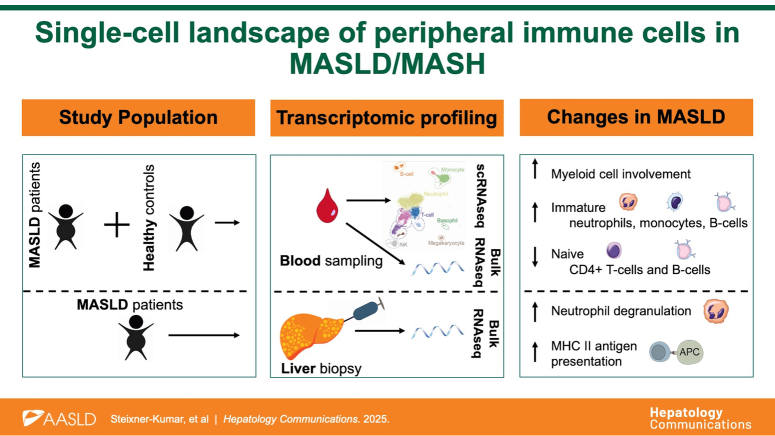

## INTRODUCTION

Metabolic dysfunction–associated steatotic liver disease (MASLD) and the more advanced form, metabolic-associated steatohepatitis (MASH), are increasingly prevalent conditions with significant clinical relevance.^1^ As now widely accepted,^2^ we use the nomenclature and criteria of MASLD and MASH instead of the previously used terms NAFLD and NASH. In general, MASLD is characterized by liver fat accumulation and in its progressive state in MASH with subsequent liver inflammation. The metabolic dysregulation is caused by cardiometabolic risk factors like type 2 diabetes and obesity and can lead to further disease worsening, i.e., liver fibrosis, cirrhosis, and death.^3^ Despite their high prevalence, there are currently only limited treatment options available.^4^ Although the molecular understanding of immunological processes for MASLD/MASH is growing (for a review, see the study by Huby et al^5^,^6^,^7^), little is known about the peripheral immune landscape of these patients. A deeper investigation of peripheral immune cells using state-of-the-art single-cell omics technologies is crucial because peripheral immune cells may act as systemic drivers of the disease since peripheral immune cells like T cells, B cells, monocytes, and neutrophils are recruited to the liver in the disease.^5^

Bulk and single-cell RNA-sequencing (RNA-seq) represent powerful tools to investigate disease-associated changes in the transcriptomic regulation at different scales as demonstrated previously for MASLD/MASH^8^,^9^ and other chronic liver diseases.^9^,^10^ In particular, single-cell RNA sequencing (scRNA-seq) of peripheral immune cells provides unprecedented molecular resolution, allowing for the identification of distinct immune cell subsets and their functional states, pivotal in understanding disease progression and developing targeted treatments. However, to the best of our knowledge, a comprehensive single-cell analysis of peripheral immune cells from histologically characterized patients with MASLD/MASH has not yet been reported.

For the present study, fresh native biopsies and blood samples from 22 prospectively enrolled and histologically characterized patients with MASLD and MASH, as well as blood samples from 14 gender-matched healthy control subjects, were collected for omics analysis. We leverage the power of bulk RNA-seq from paired blood and liver biopsy samples from the same human patients, in conjunction with blood scRNA-seq from patients and healthy controls, to gain insights into the immune response in MASLD and early MASH (fibrosis stage > F2). By integrating these complementary techniques, we aim to elucidate modulations of peripheral immune cells in disease, paving the way to identify new immune cell type–specific pathological mechanisms for novel therapeutic interventions.

## METHODS

### Human Study

#### Study design

The study has been approved by the ethical committee of Ulm University Hospital (#330/20 FSt/TR) and was conducted in accordance with the declarations of Helsinki﻿ and Istanbul. Patient subjects gave their written consent to participate in this study and have been recruited prospectively based on noninvasive metabolic-associated fatty liver disease/MASH screening. As inclusion criteria, we enrolled patients with suspected MASLD/MASH according to the 2024 multisociety Delphi consensus patient inclusion criteria.^2^ Although, at the beginning of the study, the definitions for metabolic liver disease differed from the later defined Delphi consensus, all patient cases were retrospectively checked and fulfilled the new criteria. In some cases, we assessed additional data from referring physicians or hospitals to verify the cardiometabolic criteria for adults (eg, lipid-lowering treatment). For subgroup stratification, we then applied histology-derived NAFLD activity (NAS) score. All patients gave their informed consent to participate in the study and to donate blood and liver tissue for omics analysis. Healthy subjects gave their informed consent and donated blood samples for comparative analysis to patients with MASLD/MASH.

#### Sample collection

Liver tissue from biopsies (1.4×10–22 mm) was preserved on fresh ice for further processing. Blood samples were collected at the volume of 40 mL and were transferred to tubes containing Na-Heparin (8 mL), K3-EDTA (30 mL), and Na-Citrat (2 mL). The samples were stored on ice until further processing.

#### Histology and imaging

MASLD/MASH disease state was assessed by liver histology according to the study by Kleiner et al.^10^,^11^,^12^ We used the NAS score to infer the disease severity and indicate the degree of liver inflammation to distinguish between MASLD and MASH (NAS>4).

Shear wave elastography, shear wave dispersion, and attenuation imaging (ATI) measurements, as described before,^13^ have been included for noninvasive diagnostics.

In order to focus on early inflammation in MASLD/MASH, patients with advanced fibrosis >F2 have been excluded. All included subjects were stratified with regard to the composite NAS score, i.e., summing degree of steatosis (0–3), lobular inflammation (0–3), hepatocyte ballooning (0–2), and fibrosis (0–4).^14^ Each patient was classified as low NAS (NAS<3), medium NAS (3–4), or high NAS (>4). For comparative cross-sectional omics analysis, we included balanced group sizes of 8 low NAS, 7 medium NAS, and 7 high NAS patients. Whenever possible, blood and liver samples were collected from each patient. For one of the 22 patients, only a liver biopsy sample, but no blood sample was available. We also included blood samples from gender and age-matched 14 healthy controls in the study.

#### Sample processing

White Blood cells were isolated by centrifugation of K3-EDTA blood samples and treated with erythrocyte lysis buffer. After white blood cell (WBC) isolation from blood, RNA has been isolated using RNeasy Mini Kit (Qiagen) according to the manufacturer’s instructions.

Approximately 2 mg of liver biopsies were used for the isolation of RNA. Samples were kept in 100 μL of RNAlater buffer and saved at −80 °C for further isolation of RNA. In brief, for RNA isolation the RNeasy Lipid Tissue Mini Kit (Qiagen) was used according to manufacturer’s instructions.

#### RNAseq

For bulk mRNA-seq, 60–100 ng high-quality total RNA (RIN >8) was employed for library preparation with the NEBNext Ultra II Directional RNA Library Prep Kit for Illumina, together with the NEBNext Poly(A) mRNA Magnetic Isolation Module (see Supplemental Materials and Methods for details, http://links.lww.com/HC9/B954). The final mRNA-seq libraries were normalized on the MicroLab STAR (Hamilton), pooled, and sequenced on an Illumina NovaSeq. 6000 with 2×100 bp length with a sequencing depth of ~34 million Pass-Filter reads per sample.

For scRNA-seq, isolated WBCs were resuspended in an ice-cold buffer (1xPBS + 0.04% bovine serum albumin) and filtered (40 µm, Partec). Cell concentration, viability, and aggregate were determined with the NucleoCounter NC-3000 (Chemometec). High-quality single-cell suspensions were employed for scRNA-seq library preparation with the Chromium Next GEM Single Cell 3’ Kit v3.1 (10x Genomics) according to the manufacturer’s instructions (Supplemental Materials and Methods for details, http://links.lww.com/HC9/B954). Final libraries were normalized, pooled, spiked in with 5% PhiX Control v3 (Illumina), and sequenced on an Illumina Novaseq 6000 at a depth of ~50,000 reads/cell with dual index, paired-end reads.

### Data analysis

#### Bulk RNA-seq analysis

Bulk RNA-seq analysis was performed as described before^15^ using limma-voom for downstream differential expression testing, lm function (stats R package) for linear model fitting, and Bioconductor package granulator^16^ for deconvolution. For cross-validation of liver signatures, we applied hierarchical clustering and tested for pairwise enrichment of fibrosis-associated genes derived from previously published RNAseq data set^9^ as described in Sauer et al^17^ using DEGreport2 (v1.30.0),^8^ pairwise enrichment versus the reference data set,^9^ visualization with ComplexHeatmap^18^ and functional Reactome pathway enrichment using clusterProfiler.^19^ For further details regarding bulk RNAseq analysis, see Supplemental Materials and Methods, http://links.lww.com/HC9/B954.

### Single-cell RNA-Seq analysis

For details regarding scRNA-seq analysis, see Supplemental Materials and Methods, http://links.lww.com/HC9/B954. In brief, demultiplexing and count matrix generation were performed with a cellranger. Quality control features are shown in Supplemental Figure S1A, http://links.lww.com/HC9/B961. Doublets were removed with scrublet.^20^ Samples were integrated with scVI, and graph-based clustering yielded 16 clusters. The distribution of samples within clusters and subclusters is shown in Supplemental Figure S1B, http://links.lww.com/HC9/B961, and Supplemental Table S1, http://links.lww.com/HC9/B955. Cell type annotation was done manually based on established marker genes. Manual annotations were cross-validated using the Python-based automatic cell type annotation tool CellTypist.^21^

Integration and subclustering were performed with Scanpy and scVI or Seurat.^22^ Subclusters were annotated manually and, for comparison, with Celltypist.

Cell type specificity scores were calculated as described in Supplemental Materials and Methods, http://links.lww.com/HC9/B954. Genes with a high cluster specificity score >0.65 were considered specific (Supplemental Table S2, http://links.lww.com/HC9/B956) and selected for enrichment analyses. Aggregated expression scores for each cell were then calculated and projected onto uniform manifold approximation and projection embeddings (Supplemental Figure S2, http://links.lww.com/HC9/B961). Single-cell–based differential expression was evaluated using the Seurat FindMarkers function. Pseudobulk differential expression results were generated with R package Libra and applying edgeR’s likelihood ratio test. The subset of CD14+ monocytes that were enriched in patients with MASLD/MASH was selected using Seurat CellSelector tool. The R packages DAseq^23^ and Scissor were used to perform differential abundance testing and identification of phenotype-associated cells, respectively. Trajectory analysis was performed with Monocle3.^24^

### General analysis

All analyses were done in Python or R. Multiple testing was corrected by Bonferroni (scRNA-seq differentially expressed gene [DEG] analysis) or Benjamini-Hochberg (all others) method if not indicated otherwise. Gene-set enrichment analyses (GSEA) were conducted according to the study by Korotkevich et al.^25^ For details regarding statistical tests applied, see Supplemental Materials and Methods, http://links.lww.com/HC9/B954.

### Data availability

Bulk and single-cell RNASeq raw data and the preprocessed expression matrices have been deposited in the Gene Expression Omnibus under accession codes GSE267031 (bulk liver), GSE267032 (bulk white blood cells), GSE267033 (single white blood cells), and GSE267195 (superseries).

## RESULTS

### Phenotypic characterization and clinical pathology of the study cohort

**FIGURE 1:**
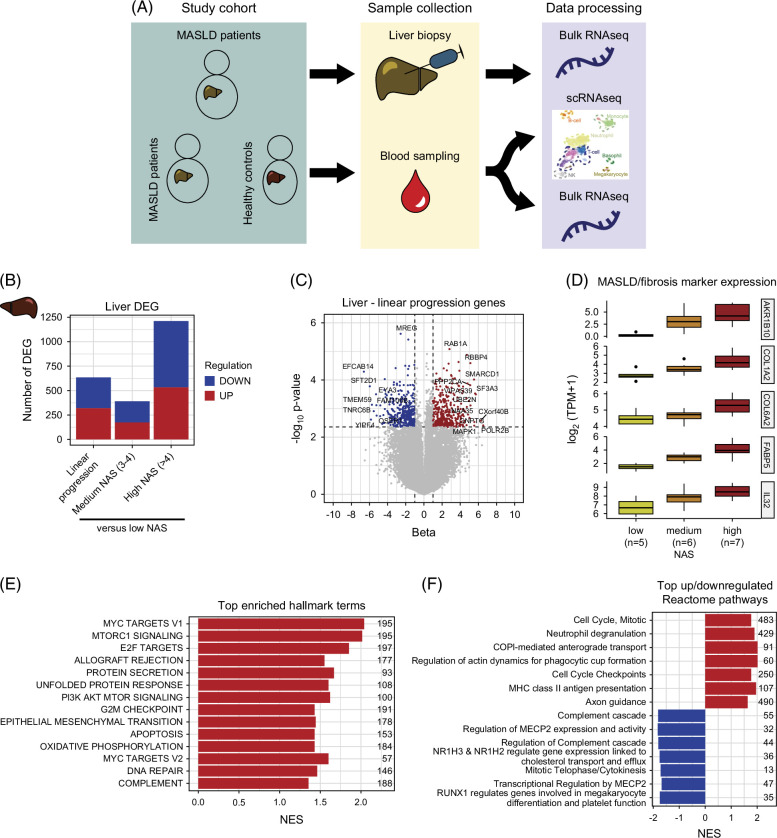
Differential expression and enrichment results from liver bulk RNAseq. (A) Study setup and data collection. (B) Number of up/downregulated DEG in a linear disease progression model (FDR<0.1, |beta|>1) and 2-group comparisons against patients with low NAS (*p*_adj_<0.05, |logFC|>0.25) (C) Volcano plot showing genes with a linear increase across MASLD severity. Significant genes with |beta|>5 are labeled. (D) MASLD and fibrosis biomarker expression increases with NAS (all FDR <0.1). (E) Significantly enriched hallmark gene sets (set size on the right) in the linear disease progression model ordered by *p* value. (F) Top 7 up/downregulated Reactome terms (set size on the right) in the linear disease progression model. Abbreviations: DEG, differentially expressed gene; MAFLD, metabolic-associated fatty liver disease; MASLD, metabolic-associated steatotic liver disease; MHC, major histocompatibility complex; NAS, NAFLD activity score; NES, normalized enrichment score.

A total of 22 patients with MASLD/MASH and 14 healthy controls were included in the study. Summary statistics of clinically relevant data from all human subjects are given in Table 1 (for summary statistics per omics data set, see Table S1, http://links.lww.com/HC9/B955). Patients and controls were matched for age (*p*=0.10) and sex (*p*=0.73). All patients underwent liver biopsy to confirm the diagnosis of MASLD/MASH and to assess the disease stage. In addition, blood samples were collected from patients and healthy controls (Figure 1A). As shown in Table 1, body mass index as well as blood levels of liver enzymes gradually increased from controls to patients with low NAS and further increased in patients with medium and high NAS. There was no significant correlation between NAS score and fibrosis stage (r=0.4, *p*=0.06). Type 2 diabetes was absent in patients with low and medium NAS but present in 4 of 7 patients with high NAS. The prevalence of hypertension was generally low in all groups. Noninvasive imaging readouts shear wave elastography (point shear wave elastography), shear wave dispersion, and ATI (liver fat quantification via attenuation imaging) were significantly increased in patients versus healthy controls (*p*=0.03, 0.006, and 0.005, respectively).

**TABLE 1:** Overview of human sample donors

Variable	Healthy control	Low NAS (<3)	Medium NAS (3-4)	High NAS (>4)	Total
Sex female (%)	8 (57.1)	4 (50.0)	2 (28.6)	4 (57.1)	18 (50.0)
Age years mean (min-max)	35.7 (22.0–56.0)	35.1 (18.0–57.0)	52.9 (34.0–71.0)	44.1 (29.0–70.0)	(-)
BMI mean (min-max)	22.7 (18.7–26.0)	25.1 (20.8–32.6)	27.3 (15.8–33.8)	29.3 (22.3–36.6)	(-)
T2D no (%)	10 (71.4)	8 (100.0)	6 (85.7)	3 (42.9)	27 (75.0)
T2D yes (%)	0	0	0	4 (57.1)	4 (11.1)
T2D NA (%)	4 (28.6)	0	1 (14.3)	0	5 (13.9)
Hypertension no (%)	9 (64.3)	7 (87.5)	5 (71.4)	5 (71.4)	26 (72.2)
Hypertension yes (%)	1 (7.1)	1 (12.5)	2 (28.6)	2 (28.6)	6 (16.7)
Hypertension NA (%)	4 (28.6)	0	0	0	4 (11.1)
AST mean (min-max)	20 (5–56)	70 (29–146)	73 (25–108)	140 (43–285)	(-)
ALT mean (min-max)	24 (14–50)	48 (27–76)	63 (33–121)	112 (43–217)	(-)
GGT mean (min-max)	22 (10–42)	110 (23–342)	294 (43–571)	505 (61–2544)	(-)
SWE mean (min-max)	4.9 (4.3–5.7)	7.8 (3.7–19.6)	7.1 (4.3–14.6)	6.5 (3.7–8.1)	(-)
SWD mean (min-max)	13.3 (11.8–15.7)	15.6 (10.9–21.6)	15.2 (12.0–21.0)	14.3 (12.8–18.0)	(-)
ATI mean (min-max)	0.6 (0.5–0.8)	0.7 (0.5–0.8)	0.7 (0.6–0.9)	0.9 (0.6–1.0)	(-)
Fibrosis F0 (%)	– (0)	3 (37.5)	0	14.3% (1)	4 (11.1)
Fibrosis F1 (%)	– (0)	4 (50.0)	2 (28.6)	2 (28.6)	8 (22.2)
Fibrosis F2 (%)	– (0)	1 (12.5)	5 (71.4)	4 (57.1)	10 (27.8)
Fibrosis NA (%)	14 (100.0)	0	0	0	14 (38.9)
N Multi-OMICs WCB-SC|WBC-Bulk|Liver-Bulk	6|14|0	2|8|5	4|6|6	3|7|7	15|35|18

*Note:* Summary statistics of clinically relevant variables from patient and healthy control groups included in the present study. For summary statistics per omics assay sample, please see Supplemental Table S1, http://links.lww.com/HC9/B955.

Abbreviations: ATI, liver fat quantification via attenuation imaging; BMI, body mass index, T2D prevalence of type-2 diabetes; n, total number of subjects; NAS, NAFLD activity score; SC, single cell; SWD, shear wave dispersion; SWE, shear wave elastography; WBC, white blood cell.

### Liver expression profiles in MASLD

In the liver, RNA-seq comparisons of patients with medium or high NAS to patients with low NAS yielded 388 (56% upregulated) and 1212 (56% upregulated) differentially expressed genes (DEG, *p*_adj_<0.05, logFC>0.25), respectively (Supplemental Table S3, http://links.lww.com/HC9/B957). Six hundred thirty-two genes showed progressively increased (~50%) or decreased (~50%) expression with increasing NAS (Figure 1B, Supplemental Table S3, http://links.lww.com/HC9/B957). Top progressively regulated genes included *MREG*, *RAB1A*, *SMARCD1*, *SF3A3*, *ILF2*, and *RAN* (Figure 1C, Supplemental Table S3, http://links.lww.com/HC9/B957). Furthermore, known MASLD-associated and fibrosis-associated genes showed a stepwise increased expression with increasing MASLD severity (Figure 1D). GSEA of hallmark terms showed upregulation of *MYC* target genes, elevated epithelial to mesenchymal transition as well as apoptosis (Figure 1E). Additional Reactome pathway GSEA showed positive enrichment for genes involved in neutrophil degranulation and major histocompatibility complex (MHC) II antigen presentation. Negative enrichment was observed for *MECP2* expression, activity, and regulation, and *NR1H2/3* (also known as Liver X receptors) regulated gene expression linked to cholesterol transport and efflux. Importantly, while “typical” fibrosis pathways were not enriched among disease progression genes, *MECP2*, and liver X receptors have both been linked to lipid metabolism and fatty liver disease.^26^,^27^

A hierarchical cluster analysis of all DEGs (n=908) yielded 6 distinct gene clusters. Among the main 3 clusters (Supplemental Figure S3A, http://links.lww.com/HC9/B961), cluster 1 shows a positive correlation with the NAS group (upregulated with disease), whereas cluster 2 shows a negative correlation with the NAS group (downregulated in disease). Functional annotation by GSEA revealed that cluster 1 is enriched for genes responsible for response to metal ions as well as complement cascade genes and that cluster 2 is mostly enriched for cell division and TCA cycle-relevant pathway genes (Supplemental Figure S3B, http://links.lww.com/HC9/B961, for complete gene enrichment results, see Table S3, http://links.lww.com/HC9/B957). For comparison and cross-validation, we tested the major gene signatures obtained with the present study for the enrichment of fibrosis-associated genes observed in a second larger study, including patients with advanced fibrosis and stratified by fibrosis stage^9^ (pairwise cluster enrichment analysis for similar analysis, see also Sauer et al^17^). The results are shown in Table S3, http://links.lww.com/HC9/B957. Accordingly, we observe the highest enrichment of gene signatures increased with fibrosis in the reference study in cluster 2 of our study, which represents genes that are increased with inflammation. Conversely, gene signatures negatively correlated with the fibrosis stage in the reference study are highest enriched in cluster 1 in our study, which represents genes that are decreased with the NAS stage (Supplemental Figure S3C, http://links.lww.com/HC9/B961).

### Increased myeloid cell involvement in the blood of patients with MASLD

**FIGURE 2:**
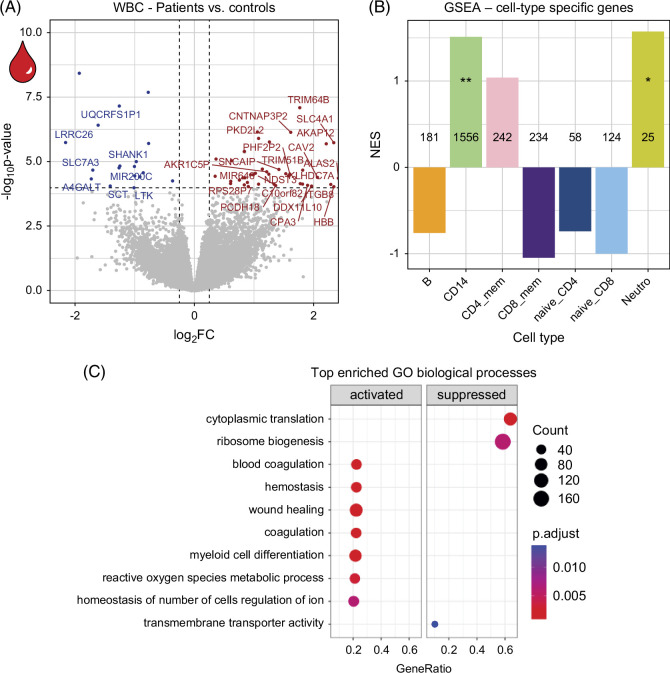
Differential expression and enrichment results from blood bulk RNAseq. (A) Volcano plot showing DEG in WBC RNAseq of patients versus controls. Significant genes with |logFC|>1 are labeled. (B) GSEA of cell type–specific genes in DE analysis comparing patients versus controls. Numbers indicate gene set sizes. **p_adj_<0.05, *p<0.05. (C) Top 10 biological processes in GO enrichment analysis of DE results (patients vs. controls). Abbreviations: GO, gene ontology; GSEA, gene-set enrichment analysis; NES, normalized enrichment score; WBC, white blood cell.

In contrast to the strong transcriptional changes observed in the bulk liver, RNA-seq of WBC did not reveal any transcriptional changes that were linearly associated with increasing NAS (all false discovery rate>0.1). A comparison of patients stratified by NAS to healthy controls revealed a progressive decrease in the number of DEG with increasing NAS (Supplemental Figure S3D, http://links.lww.com/HC9/B961). Overall, 63 genes (71% upregulated) were differentially expressed in patients versus healthy controls (Supplemental Table S3, http://links.lww.com/HC9/B957). Figure 2A shows a volcano plot of all DEG. GSEA of cell type–specific genes (derived from corresponding blood scRNA-seq data, see Supplemental Figure S2, http://links.lww.com/HC9/B961) showed positive enrichment for CD14+ monocyte and neutrophil-specific genes among differential expression (DE) results (Figure 2B). In addition, results indicated activation of myeloid cell differentiation, reactive oxygen species, coagulation as well as wound healing and hemostasis. Transcriptional pathways for cytoplasmic translation and ribosome biogenesis were downregulated (Figure 2C).

### Landscape of peripheral immune cells in MASLD

**FIGURE 3:**
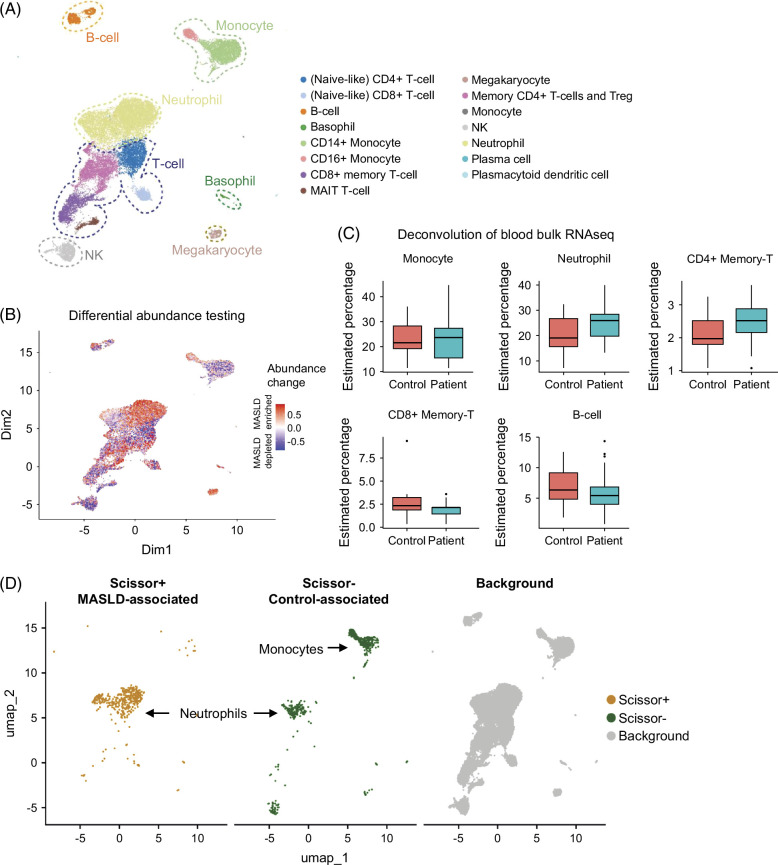
Whole blood single-cell atlas of patients with MASLD (n=9) and healthy controls (n=6). (A) Landscape of immune cell populations in the blood. (B) Differential abundance testing in healthy controls vs. patients across all cell types. (C) Deconvolution estimates of major immune cell populations from blood bulk RNAseq. All comparisons are nonsignificant (*p*>0.05, Welch test). (D) Cell populations associated with the patient (Scissor+ cells) or control status (Scissor−). Abbreviations: MASLD, metabolic-associated steatotic liver disease; NK, natural killer.

After data analysis and quality control of scRNA-seq we obtained transcriptomic profiles from 29,890 single immune cells. Figure 3A depicts the landscape of all major peripheral blood immune cell populations in patients with MASLD (n=9) and controls (n=6). Integration, dimensionality reduction, and unsupervised clustering revealed 15 major immune cell populations. Each cell cluster was subsequently annotated by using known cell type–specific marker genes from the literature (Supplemental Figure S1C, http://links.lww.com/HC9/B961, Table S4, http://links.lww.com/HC9/B958). Annotations were cross-validated with the automatic cell type annotation tool CellTypist (Supplemental Figure S4, http://links.lww.com/HC9/B961). Differential abundance testing (DAT; Figure 3B) on the scRNA-seq data set was complemented by deconvoluting the WBC bulk RNA-seq data (Figure 3C). This revealed for the first time paired single-cell/bulk RNA-seq derived insights into immune cell type abundance changes in the blood of patients with MASH/MASLD in comparison to healthy controls. In general, both methods showed consistent trends within major cell populations, indicating overall enrichment of neutrophils, and depletion of CD8+ memory T cells in MASLD/MASH versus healthy controls. The absence of significant changes in deconvolution of major immune cell populations can be explained by the presence of bidirectional abundance changes within these larger populations, as depicted in Figure 3B, that point to differential responses of specific subpopulations. As another integrative method for bulk and single-cell data, we applied Scissor^28^ to identify specific cell populations that are associated with either MASLD subjects or healthy controls. This approach identified a subset of neutrophils as positively associated with MASLD and another subset of neutrophils associated with healthy controls. Moreover, a subset of monocytes showed an association with control status. In agreement with bulk RNA-seq analyses (Figure 2), these findings point to an enhanced involvement of peripheral myeloid cells in MASLD.

Given the heterogenous picture of abundance changes and phenotype-associations within major cell types, we hypothesized that the observed heterogeneity is driven by differential responses of cellular subtypes in MASLD. Thus, we next sought to further investigate each major cell type in-depth to unravel the complexity of the peripheral immune landscape in MASLD.

### Immature circulating neutrophils are associated with MASLD

**FIGURE 4:**
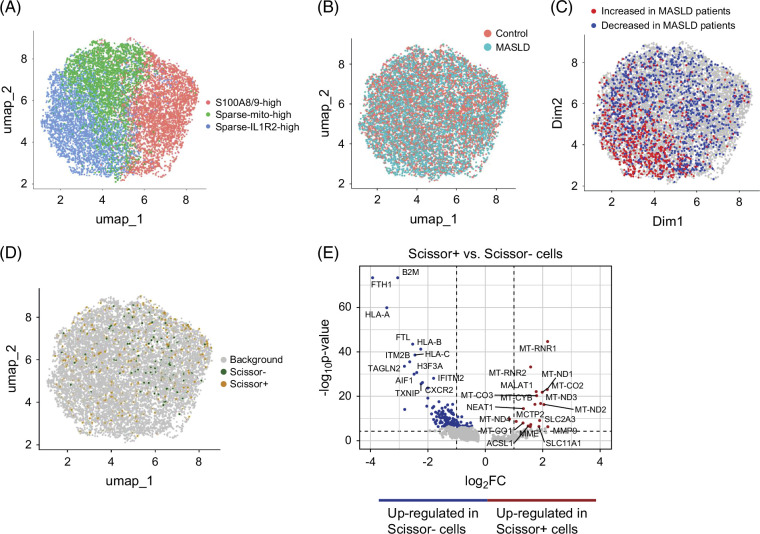
Neutrophil subpopulation shifts in healthy controls and patients with MASLD. (A) UMAP embedding showing subpopulations of neutrophils. (B) Distribution of cells from control and MASLD patient samples. (C) Differential abundance testing (cutoff ± 0.7) showing MASLD depleted/enriched subpopulations. (D) Projection of Scissor+ (MASLD-associated) and Scissor− (control-associated) cells as identified in Figure 3D onto subclustered neutrophil data. (E) Differential expression between Scissor+ (MASLD-associated) and Scissor− (control-associated) neutrophils. Abbreviations: MASLD, metabolic-associated steatotic liver disease; UMAP, Uniform Manifold Approximation and Projection.

Neutrophils were extracted from the full scRNA-seq data set, reintegrated, and reclustered to investigate neutrophil subpopulations in detail. This approach yielded 3 subpopulations that were characterized by the expression of specific marker genes and/or mean gene expression level (Figure 4A, for subcluster markers, see Supplemental Table S5, http://links.lww.com/HC9/B959). DAT indicated enrichment of a population of *IL1R2*-high neutrophils in patients with MASLD that further exhibited elevated expression of, eg, *PHACTR1*, *NEAT1*, *MALAT1*, *CPQ*, *CELF2,* and *CFLAR*. The remaining 2 cell clusters were found to be depleted in patients (Figures 4B, C). The projection of phenotype-associated neutrophils, as identified in Figure 3D, onto the neutrophil subclusters showed that these phenotype-associated cells (Figure 4D) do not clearly correspond to one of the three single subpopulations shown in Figure 4A. To further characterize these MASLD-associated (Scissor+) versus control-associated cells (Scissor−), we generated differential expression profiles of these 2 populations (Figure 4E, for pseudobulk-based DE results, see Supplemental Figure S5A, http://links.lww.com/HC9/B961 and Supplemental Table S6, http://links.lww.com/HC9/B960) and observed that the former was composed of 2 neutrophil populations previously identified by Wigerblad et al^29^: immature neutrophils with high expression of *MME*, *MMP9*, *FCN1*, *CAMP*, *CYBB*, and *CST3* (corresponding to Nh0 neutrophils in Wigerblad et al^29^) as well as mature neutrophils with high expression of *MALAT1* and *NEAT1* (corresponding to Nh2 neutrophils in Wigerblad et al^29^). Interestingly, *MMP9* expression in the liver of patients correlated positively (r=0.63, *p*=0.005) with ATI measurements, performed to evaluate liver steatosis. In comparison to Scissor− neutrophils, disease-associated (Scissor+) neutrophils had a high expression of mitochondrial and ribosomal genes, which could be a biological correlate of immature neutrophils,^30^ indicate compromised quality of these cells or a combination thereof, i.e., immature neutrophils express mitochondrial and ribosomal genes more strongly, because they are more vulnerable to the protocol applied. Considering this, findings related to this population should be interpreted with caution. Control-associated neutrophils were of a transitional state (maturity between immature and fully mature neutrophils) with elevated expression of *AIF1*, *CXCR2*, and *TXNIP*, representing the most common type of circulating neutrophils in humans (corresponding to Nh1 neutrophils in Wigerblad et al^29^).

### Increase of myeloid-derived suppressor cells in MASLD

**FIGURE 5:**
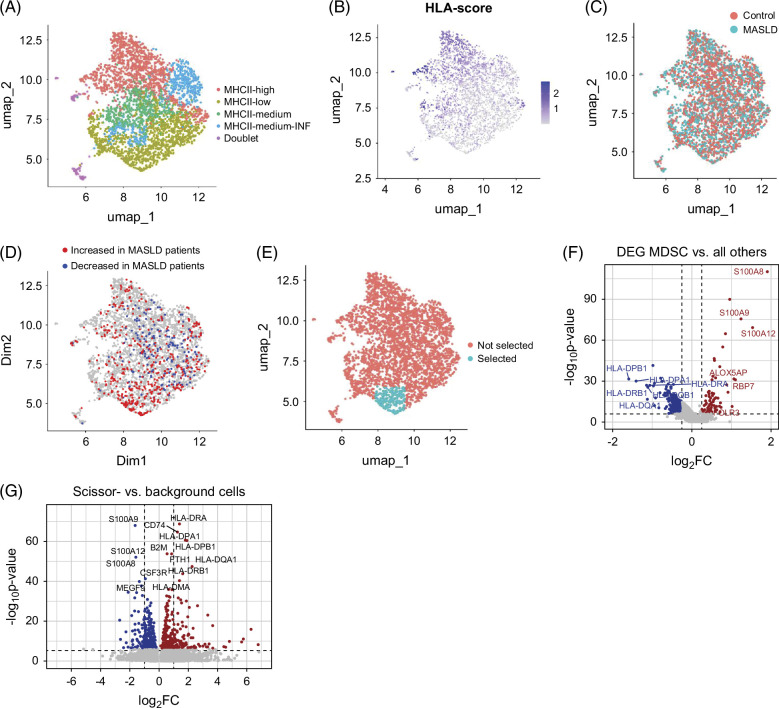
CD14+ monocyte subpopulation shifts in healthy controls and MASLD. (A) UMAP embedding showing subpopulations of CD14+ monocytes. (B) Aggregated expression of HLA genes. (C) Distribution of cells from control and MASLD patient samples. (D) Differential abundance testing (cutoff ±0.7) showing MASLD depleted/enriched subpopulations. (E) Selection of MASLD-enriched subpopulation using Seurat CellSelector tool. (F) Volcano plot showing DEG between the MASLD-enriched subpopulation as selected in (E), characterized as MDSCs, and all other cells. Positive log2-fold changes indicate upregulation in the selected population. Genes with an|log2FC|>1 are labeled. (G) Volcano plot showing DEG between Scissor- and background CD14+ monocytes as identified in Figure 3D. Abbreviations: DEG, differentially expressed genes; HLA, human leukocyte antigen system; MASLD, metabolic-associated steatotic liver disease; MDSC, myeloid-derived suppressor cell; MHC, major histocompatibility complex; UMAP, Uniform Manifold Approximation and Projection.

Next, we analyzed CD14+ monocytes in detail. Subclustering revealed four distinct monocyte subpopulations (Figure 5A, for subcluster markers, see Supplemental Table S5, http://links.lww.com/HC9/B959). Subpopulations could be characterized by their expression of MHC II complex genes (Figure 5B). Comparison of the distribution of patient and control cells and DAT showed a mild depletion of cells with medium expression of MHC II genes in MASLD and a pronounced increase in abundance of a small subset of cells with low MHC II expression in patients with MASLD (Figure 5C/D). This subpopulation was selected using Seurat CellSelector tool for further characterization (Figure 5E). Contrasting the selected population with all remaining CD14+ monocytes in DE analysis uncovered high expression of *S100A8*, *S100A9,* and *S100A12* and diminished expression of MHC II genes, including HLA-*DPB1*, *HLA*-*DPA1*, *HLA*-*DRB1*, and *HLA*-*DQA1* (Figure 5F, for pseudobulk-based DE results, see Supplemental Figure S5B, http://links.lww.com/HC9/B961 and Supplemental Table S6, http://links.lww.com/HC9/B960). This expression profile indicated that these cells can be characterized as a subset of myeloid cells known as myeloid-derived suppressor cells (MDSC).^31^ Notably, the expression of *S100A8*, *S100A9*, and *S100A12* in bulk liver tissue was positively correlated with ATI (*S100A8*: r=0.54, *p*=0.02; *S100A9*: r=0.64, *p*=0.004; *S100A12*: r=0.49, *p*=0.04), potentially indicating an accumulation of these cells in the liver of patients with MASLD, similar to what has been observed in a mouse model of NAFLD.^32^

In line with DA testing results, we observed that control-associated CD14+ monocytes (Scissor−) as described in Figure 3D, had higher expression of MHC II genes (Figure 5G, for pseudobulk-based DE results, see Supplemental Figure S5C, http://links.lww.com/HC9/B961 and Supplemental Table S6, http://links.lww.com/HC9/B960) as compared to the rest of the population (“background cells”).

### Increase of transitional B cells in MASLD

**FIGURE 6:**
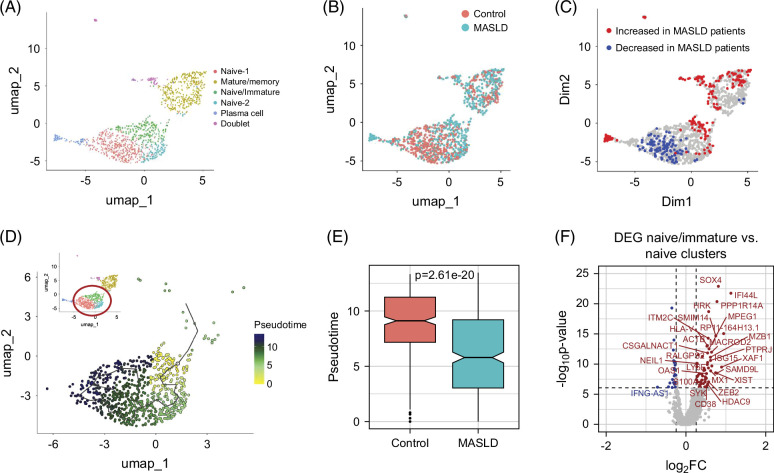
B-cell subpopulation shifts in healthy controls and patients with MASLD. (A) UMAP embedding showing subpopulations of B cells and plasma cells. (B) Distribution of cells from control and MASLD patient samples. (C) Differential abundance testing (cutoff ±0.7) showing MASLD depleted/enriched subpopulations. (D) Trajectory analysis of the immature/naïve and naive B-cell populations. Inset shows the selected target population circled in red. (E) Average pseudotime of cells in the selected population comparing healthy controls and patients with MASLD. Welch *t* test applied. (F) Volcano plot showing DEG between naïve/immature and naive clusters. Positive log2-fold changes indicate upregulation in the naive/immature population. Genes with an|log2FC|>0.5 are labeled. Abbreviations: DEG, differentially expressed gene; MASLD, metabolic-associated steatotic liver disease; UMAP, Uniform Manifold Approximation and Projection.

The analysis of myeloid lineage cells was followed by a detailed investigation of lymphoid cells. First, we performed an analysis of B cells in MASLD. B cells clustered into 5 distinct B-cell populations (Figure 6A) that could broadly be divided into immature, naive, and mature B cells as well as plasma cells. Expression patterns of major marker genes that were used to differentiate the different subpopulations are shown in Supplemental Figure S6, http://links.lww.com/HC9/B961 (full list of subcluster markers in Supplemental Table S5, http://links.lww.com/HC9/B959). We observed an enrichment of immature/naive B cells in patients with MASLD and a depletion of cells in the naive-1 subpopulation. Further, there was also a partial increase in mature B cells in patients (Figures 6B, C). To further confirm the shifted maturation process in non-mature B cells, we performed a trajectory analysis to infer the maturity state of each cell (Figure 6D). Comparing the inferred “age/maturity” of the individual B-cell cluster showed highly significant differences between the three nonmature B-cell subpopulations (Kruskal-Wallis test, *p*=9.84e-50). As expected, the immature/naive cluster had the lowest inferred “age/maturity” followed by the naive-2 cluster. Naive-1 cluster represented the most mature naïve B cells. To validate this finding in a clustering-independent way, we calculated the average pseudotime in patients versus healthy controls across non-mature cell clusters (Figure 6E). Consistent with the pattern observed in DAT, we found pseudotime to be significantly lower in the MASLD group (reduced maturated naive cells, increased immature cells). Subsequently, we investigated the population of immature/naive B cells by DE analysis. Accordingly, the immature/naive B cells exhibited a signature of strongly upregulated genes, including *SOX4*, *MZB1*, and *CD38* (for pseudobulk-based DE results, see Supplemental Figure S5D, http://links.lww.com/HC9/B961 and Supplemental ble S6, http://links.lww.com/HC9/B960). Amongst other markers, this suggested that these cells are transitional B cells, which represent a class of immature B cells that undergo maturation to either become follicular B cells or marginal zone B cells and that can be detected in human peripheral blood.^33^ Further, these cells also showed strong expression of interferon response genes, such as *ISG15*, *MX1*, and *IFI44L*.

### Depletion of naive-like CD4+ T cells in MASLD and enrichment of Tregs

**FIGURE 7:**
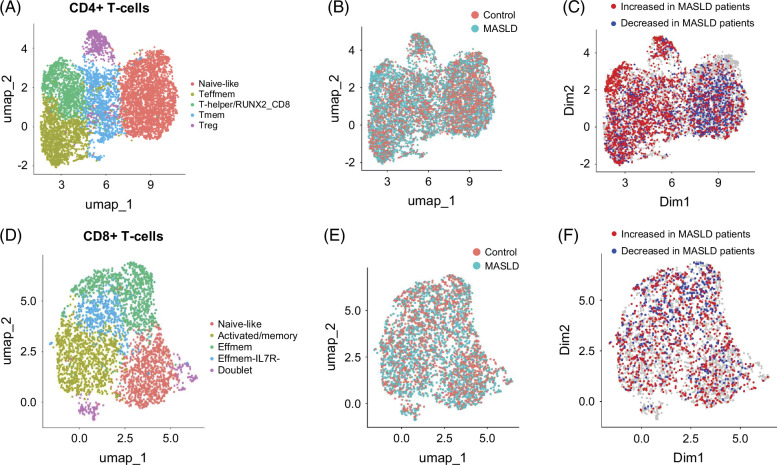
T-cell subpopulation shifts in healthy controls and patients with MASLD. (A) UMAP embedding showing subpopulations of CD4+ T cells. (B) Distribution of CD4+ T cells from control and MASLD patient samples. (C) Differential abundance testing (cutoff ± 0.7) showing MASLD depleted/enriched CD4+ T-cell subpopulations. (D) UMAP embedding showing subpopulations of CD8+ T cells. (E) Distribution of CD8+ T cells from control and MASLD patient samples. (F) Differential abundance testing (cutoff ± 0.7) showing MASLD depleted/enriched CD8+ T-cell subpopulations. Abbreviations: MASLD, metabolic-associated steatotic liver disease; UMAP, Uniform Manifold Approximation and Projection.

Finally, we explored T cells in MASLD. CD4+ T cells were partitioned into 5 subpopulations (Figure 7A, subcluster markers in Supplemental Table S5, http://links.lww.com/HC9/B959). The largest population was formed by naive-like T cells that showed depletion in patients with MASLD (Figure 7B/C). Effector-memory, memory, and T-helper cells showed a mixed pattern of abundance changes, with enrichment in MASLD prevailing. Regulatory T cells (Tregs) were found to be increased in MASLD (Figure 7C). An observed enrichment of *RUNX2*+ CD8+ cells (left-upper portion of T-helper/RUNX2_CD8 cluster) in MASLD could not be interpreted, as this population was primarily derived from a single patient.

Among CD8+ T cells, we identified 4 specific subpopulations, ranging from naive-like to effector memory and activated plus memory cells (Figure 7D, subcluster markers in Table S5, http://links.lww.com/HC9/B959). As shown in Figures 7E, F, there was no strong and unidirectional abundance change in any CD8+ T-cell population.

## DISCUSSION

In summary, we present a comprehensive characterization of the peripheral immune cell compartment of MASLD based on omics assays. Our findings highlight the potential of paired scRNA-seq and bulk RNA-seq for obtaining cell and cell type–specific information. Whereas a broader exploration of the bulk and scRNA-seq data yielded the first indications of a prominent involvement of neutrophils and monocytes in MASLD, cell type subclustering allowed a more detailed picture of the behavior of different peripheral immune cell subsets in disease. Our findings provide unprecedented insights into peripheral immune subpopulations in MASLD and help to gain a better understanding of the immune landscape in this disease. Ultimately, these findings may point to potential new therapeutic targets for the treatment of MASLD.

Bulk DE analysis showed strong disease-associated shifts in liver biopsies of patients. We could show that known MASLD and fibrosis marker genes were upregulated with disease progression in the liver in addition to known disease-relevant pathways such as apoptosis and epithelial mesenchymal transition. This observation, in conjunction with the present upregulation of the extracellular matrix organization pathway, is particularly interesting, considering that our cohort is composed of patients with no or low-grade fibrosis.

Additionally, pathway analyses pointed to a gradual upregulation of target genes of the proto-oncogene *MYC* with disease progression. This is in line with previous reports of an enhanced expression of c-MYC in the liver of patients with MASLD and MASLD-related HCC.^34^ Along these lines, many of the top disease progression genes have previously been associated with liver cancer/HCC development and prognosis, eg, *RAN*,^35^ *SF3A3*,^36^ *SMARCD1*,^37^ *RAB1A*.^38^ Moreover, pathway analyses revealed a suppression of MECP2 signaling with increasing disease severity. In animal studies, *MECP2* deletion led to the development of fatty liver,^27^ supporting a potential role of this gene in MASLD development and progression. Another pathway with potential disease relevance includes *NR1H2/NR1H3* (alias Liver X receptors), a gene family that regulates lipid homeostasis and inflammation. Our findings indicate a suppression of this pathway with increasing disease severity, while *NR1H3* has previously been shown to be increased in the liver of patients with NAFLD as compared to healthy controls.^26^ A possible explanation might be that while this pathway is globally upregulated in patients, there is a progressive downregulation/normalization with increasing disease severity. Alternatively, the increased presence of its oxysterol ligands—as has been previously reported in NASH^39^—could lead to a negative feedback mechanism that results in a gradual decrease in transcription of the receptor during disease progression.

Another interesting finding was the positive enrichment of immune-specific functions among disease progression genes, including neutrophil degranulation, MHC class II antigen presentation, and phagocytic cup formation, indicating an important involvement of myeloid cells in MASLD progression in the liver (compare also to enhanced antigen presentation via MHC class II in HBV-related cirrhosis patients^40^). Whereas the disease-related transcriptional modulation in the blood was weak in comparison to the liver, the importance of myeloid cells was further highlighted by the enrichment of blood DE results for CD14+ monocyte and neutrophil-specific genes as well as the activation of myeloid cell differentiation pathways. Further, these findings were supported by Scissor analysis of blood scRNA-seq data, showing a selective association of neutrophils and monocytes with patient and/or control status.

So far, the role of neutrophils in MASH/MASLD has not been extensively studied by omics analysis before. In-depth evaluation of case and control-associated neutrophils revealed that a population of immature circulating neutrophils is associated with MASLD. Immature circulating neutrophils have been shown to increase sepsis and severe inflammation,^41^ but to date have not been linked to MASLD and MASH. In contrast, the presence of another immature immune cell type, namely MDSC, has been previously reported to be increased in the blood of two liver-related diseases, HCC, and chronic hepatitis C,^42^ and was found to be enriched in the blood of patients with MASLD in our study. MDSC can suppress T-cell function, thereby limiting inflammatory responses and maintaining immunotolerance and homeostasis.^43^ They have been described in various cancers, infections, autoimmune diseases as well as acute inflammation.^44^ The role of MDSC in chronic inflammation and liver disease is less understood.^42^ Yet, the liver is a main site of MDSC accumulation,^42^ and MDSC was shown to accumulate in the liver of a NAFLD mouse model.^32^ Interestingly, in our study, liver expression of MDSC markers *S100A8/S100A9/S100A12* was positively correlated with ATI, a measure of steatosis. MDSC suppresses T cells via the release of reactive oxygen species, a pathway that was also found to be activated in the blood of patients with MASLD in our study. This finding could indicate that MDSC already exerts suppressive functions in the blood of patients with MASLD and that their suppressor activity is not limited to the liver.

One more immature cell subtype, namely circulating immature/transitional B cells, displayed increased abundance in patients with MASLD. The abundance of these cells has been reported to be altered in the blood of patients suffering from immunodeficiencies or systemic lupus erythematosus,^45^,^46^ but to date, no alteration of this cell subtype has been associated with MASLD. We also observed a relative depletion of a subset of naive B cells (naive-1) in MASLD. This finding complements the recent observation by Bai et al^40^ of the depletion of naive B cells in the livers of HBV-related cirrhosis patients. Both MASLD and HBV represent chronic liver diseases triggering an inflammatory response in the liver and are linked to fibrosis, cirrhosis, and HCC.^47^ Depletion of naive B cells in the blood of patients with MASLD could also translate to a depletion of this cell type in the liver, leading to a similar situation as was observed in HBV-related cirrhosis. Similarly, naive CD4+ T cells were depleted in the blood of patients, while activated CD4+ T cells were enriched. This is in line with previous reports of changes in the peripheral immune cell compartments of patients with NAFLD/NASH.^6^

One limitation of our study is that despite extensive characterization of transcriptional changes in the liver and the blood, the question of how these changes translate to functional modulation of the immune system in MASLD/MASH remains open. Certainly, in future investigations, one valuable addition would be to translate the findings from the transcriptional to the protein level using, eg, proteomics. Furthermore, primary cell assays for major target cell populations, eg, MDSC, immature neutrophils, and B cells, are needed to test the functional consequences of the observed molecular changes. Additionally, increased sample size in terms of individuals included and cell numbers captured (scRNA-seq) would increase statistical power and thus enhance the possibility of scientific discovery and statistically account for possible confounders and comorbidities such as body mass index or diabetes type 2. Moreover, it would be desirable to match healthy controls regarding body mass index and diabetes type 2 to strengthen the results of this study. Due to the limited statistical power, the current scRNA-seq analysis only considered patient versus healthy donors and could not investigate changes related to the NAS stage. In addition to increased sample size, statistical power could be increased in future studies by applying separate protocols for peripheral blood mononuclear cells and neutrophils instead of a single protocol for total white blood cells as used in the present study. This could reduce technical inter-sample variability and increase cell capture for peripheral blood mononuclear cells and neutrophils because the currently applied scRNA-seq protocol (10X genomics v3.1) is not optimal for recovery of neutrophils which are vulnerable to technical handling. This is of particular relevance for detecting changes in the peripheral blood that are more subtle as compared to liver-related changes.

In conclusion, our study provides a comprehensive characterization of the single-cell landscape of peripheral immune cells in MASLD. Single-cell resolution and the application of paired single-cell and bulk RNA-seq analysis allows unprecedented assessment of differential cell type abundance and cell subtype identification. The most striking observation was the enrichment of immature cells (monocytes, neutrophils, B cells) and depletion of naive cells (B cells, CD4+ T cells). Future research will be needed to further shed light on the functional relevance of these changes for MASLD development and progression. Such investigations will help to find potential therapeutic opportunities targeting these cell subpopulations or specific hub genes crucial for their function. Moreover, this could open the path to the identification of new blood biomarkers for disease and disease progression. Identification of biomarkers would be of utmost importance, as this reduces the frequency and necessity of invasive liver biopsies for diagnosis and thus could provide immediate benefit for the patient.

## Supplementary Material
