# Supplementary material for: Single-cell landscape of peripheral immune cells in MASLD/MASH
Source: Hepatol Commun. 2025 Apr 21;9(5):e0643. doi: 10.1097/HC9.0000000000000643 (PMC12014121; doi:10.1097/HC9.0000000000000643)
Supplement: Supplementary file 1 [file hc9-9-e0643-s001.docx]

# SDC, Materials and Methods

# Steixner-Kumar et al. “Single-cell landscape of peripheral immune cells in MASLD/MASH”

## RNAseq

### Bulk RNAseq

For bulk mRNA-seq of patient liver biopsies and isolated WBCs, total RNA samples were quantitatively and qualitatively assessed using the fluorescence Broad Range Quant-iT RNA Assay Kit (Thermo Fisher Scientific) and the Standard Sensitivity RNA Analysis DNF-471 Kit on a 96-channel Fragment Analyzer (Agilent), respectively. All total RNA samples had a RIN >8. Total RNA input of 60-100ng was employed for library preparation with the NEBNext Ultra II Directional RNA Library Prep Kit for Illumina #E7760, together with the NEBNext Poly(A) mRNA Magnetic Isolation Module #E7490 and NEBNext Multiplex Oligos for Illumina #E7600 (New England Biolabs) as per manufacturer’s instructions. Double-stranded cDNA purification was conducted with Ampure XP beads (Beckman Coulter). Libraries were amplified with 13-14 PCR cycles. The final mRNA-seq libraries were eluted in EB Buffer (Qiagen) and were quantified by the High Sensitivity dsDNA Quanti-iT Assay Kit (ThermoFisher) on a Synergy HTX (BioTek). Libraries were assessed for size distribution and adapter dimer presence (<0.5%) by the High Sensitivity NGS DNF-474 Kit on a 96-channel Fragment Analyzer (Agilent). Libraries were normalized on the MicroLab STAR (Hamilton), pooled and sequenced on a NovaSeq 6000 (Illumina) with dual index, paired-end reads at 2 x 100 bp length with a sequencing depth of ~34 million Pass-Filter reads per sample.

### Single-cell RNAseq

For scRNA-seq, isolated WBCs were resuspended in ice-cold buffer (1xPBS + 0.04% BSA) and filtered through a 40µm Flowmi® cell filter (Partec). Cell concentration, viability, and aggregate were determined with the NucleoCounter NC-3000 (Chemometec). 15 single-cell suspensionswith an average cell viability of 93% and cell aggregate <1% were employed for scRNA-seq library preparation. Libraries were prepared with the Chromium Next GEM Single Cell 3' Kit v3.1 (10x Genomics) according to the manufacturer’s instructions (CG000315 Rev D). Briefly, ~8,250 cells were loaded into a GEM Chip G with a targeted cell recovery of ~5,000 cells and capture and GEM generation was conducted with the Chromium controller followed by reverse transcription and cDNA amplification (13 cycles) as per manufacturer’s instructions (10x Genomics). Qualitative and quantitative measurement of cDNA was conducted with the High Sensitivity NGS Fragment 1-6000bp kit on a 48-channel Fragment Analyzer (Agilent) and 1x dsDNA kit on the Qubit 4 Fluorometer (ThermoFisher), respectively. A total of 50 ng of cDNA per sample was employed for library preparation with an additional 1x SPRISelect bead clean-up (Beckman Coulter) to ensure full removal of primer and adaptor dimers prior to the final elution. Libraries were amplified with 13 PCR cycles and quantified with the 1x dsDNA kit on the Qubit 4 Fluorometer (ThermoFisher) and qualitatively assessed with the High Sensitivity NGS Fragment 1-6000bp Kit on a 96-channel Fragment Analyzer (Agilent). Final libraries were normalized, pooled, spiked in with 5% PhiX Control v3 (Illumina) and sequenced on an Illumina Novaseq 6000 at a depth of ~50,000 reads/cell with dual index, paired end reads (Read parameters: Rd1: 28 bp, Rd2: 10 bp, Rd3: 10 bp Rd4: 91bp)

## Data Analysis

### Bulk RNAseq

The bulk RNA-Seq analysis pipeline was executed as described before^1^. In brief, reads passing quality control filter were mapped against GRCh38 using STAR (v2.5.2b) aligner. Gene expression levels were quantified using RSEM (v1.3.0) and featureCounts (v1.5.1). For quality control FastQC (v0.11.5), picardmetrics (v0.2.4) and dupRadar (v1.2.2) were employed.

The limma-voom approach was used for differential expression testing. Linear disease progression models were constructed with the *lm* function (stats R package) based on voom transformed expression values. Analyses were corrected for sex, sequencing batch and age (linear models only). Deconvolution was performed with non-negative least squares (nnls) regression using the ABIS_S1 signature matrix available with the granulator^2^ bioconductor package in R.

For hierarchical clustering, all protein coding genes with significant differential expression (adjusted *p*-value ≤ 0.05) in at least one pairwise comparison against low NAS were selected. The hierarchical cluster analysis results of the fibrosis-stratified liver bulk RNAseq dataset^3^ (GSE135251), were adopted from ^4^. In short, this analysis included all genes differentially expressed in at least one pairwise comparison of F1–F4 vs. F0 (adjusted *p*-value ≤ 0.05, |log2FoldChange| ≥0.2).

Clustering was performed using DEGreport2 (v1.30.0) as described in ^5^. Only clusters with at least 20 genes were retained. Pairwise enrichment between gene clusters derived from reference data^3^ vs. our data was calculated using hypergeometric testing. Heatmaps were created with ComplexHeatmap^6^ (v2.16.0). Functional enrichment (Reactome) of clusters was evaluated using clusterProfiler^7^ (v4.2.2).

**scRNAseq**

Demultiplexing and count matrix generation were performed with cellranger v6.0.0. Reads were mapped to reference genome GRCh38.86. Quality control and clustering were performed in Python using scanpy v1.9.3 with following parameters: Cells with <30 genes or with >30% mitochondrial genes expressed were excluded. Quality control features are shown in Figure S1A. Moreover, cells were removed if they deviated from the expected ratio of number of expressed genes by total counts. Raw read counts were normalized to 10,000 counts and log-transformed. Doublets were removed using scrublet^8^. One sample with a very high cell number, was randomly downsampled to the next largest sample size (n= 3252) before integration with all remaining samples to avoid its overrepresentation. Samples were integrated with scVI based on the latent representation derived from top 6,000 highly variable genes using percentage of mitochondrial and ribosomal genes as covariates. Subsequently, a nearest neighbour graph was constructed and graph-based clustering of 29,890 cells that passed quality control was performed at resolution 0.4 yielding 16 clusters. Distribution of samples within clusters and subclusters is shown in Figure S1B and Table S1. Cell type annotation was done manually based on established marker genes and two existing neutrophil clusters were merged. Manual annotations were cross-validated using the python-based automatic cell type annotation tool CellTypist^9^ by applying the low-resolution immune cell model ('Immune_All_High.pkl’) with majority voting.

For sub-clustering, the subset of cells from the respective clusters in each sample were re-integrated based on top 2000-6000 variable features (depending on dataset size). Sample-wise integration and subclustering were performed with scanpy and scVI, except for the smallest cell type (B-cells) that was integrated using Seurat v4.3.0^10^ based on batch information. Sub-clustering was performed at resolution 0.5 or 0.6 (depending on dataset size and cell type complexity). The resulting subclusters were annotated manually using established marker genes. For comparison, annotations of subclusters was also performed using CellTypist’s high resolution immune cell model (‘Immune_All_Low.pkl’).

Cell type specificity scores were calculated as follows:

0.5+0.5*(median_target_cell_type_expression-total_median_expression)/(0.001+ median_target_cell_type_expression+total_median_expression).

Genes with a score>0.65 were considered specific (Table S2) and used for enrichment analyses. Aggregated expression scores for each cell were then calculated in Seurat using the *AddModuleScore* function and projected onto UMAP embeddings (Figure S2). The HLA-score represents the aggregated expression of following genes: *HLA-DRA*, *HLA-DPB1*, *HLA-DMA*, *HLA-DMB*, *HLA-DPA1*, *HLA-DQB1*, *HLA-DQA1*, *HLA-DRB1*. Single-cell based differential expression was evaluated using the Seurat’s *FindMarkers* function. Pseudobulk differential expression results were generated with R package Libra and applying edgeR’s likelihood ratio test (LRT). The subset of CD14+ monocytes that were enriched in MASLD/MASH patients, was selected using Seurat’s *CellSelector* tool.

The R packages DAseq^11^ and Scissor^12^ were used to perform differential abundance testing and identification of phenotype-associated cells, respectively. Scissor allows to identify phenotype-associated cells by evaluating the correlation between a cell’s expression profile and each bulk sample, followed by regression of the correlation matrix with the phenotype (patient/control) to identify phenotype-associated cells. Trajectory analysis was performed with Monocle3^13^.

### General analysis

All analyses were done in Python v3.9.5 or R v4.1.2. Multiple testing correction was performed with the Bonferroni (scRNA-seq DEG analysis) or Benjamini-Hochberg (all others) method if not indicated otherwise. Gene-set enrichment analyses (GSEA) were conducted with the fgsea package^14^ in R on the full results tables ordered by log2fold_change*-log10(p.value) or beta*-log10(p.value), respectively. Correlation analysis between gene expression and phenotypes was performed using Spearman or Pearson correlation, as appropriate. Differences between case and control group regarding phenotypical variables was evaluated using Welch’s test (continuous variables) or Fisher’s exact test (categorical variables).

1. Simon, E. *et al.* Transcriptomic profiling of induced steatosis in human and mouse precision-cut liver slices. *Sci. Data* **10**, 304 (2023).

2. Pfister, S., Kuettel, V. & Ferrero, E. *Granulator: Rapid Benchmarking of Methods for in Silico Deconvolution of Bulk RNA-Seq Data.* (2021).

3. Govaere, O. *et al.* Transcriptomic profiling across the nonalcoholic fatty liver disease spectrum reveals gene signatures for steatohepatitis and fibrosis. *Sci Transl Med* **12**, eaba4448 (2020).

4. Sauer, J. *et al.* Diverse potential of secretome from natural killer cells and monocyte-derived macrophages in activating stellate cells. *Front. Immunol.* **15**, 1232070 (2024).

5. Pantano, L. *et al.* Molecular characterization and cell type composition deconvolution of fibrosis in NAFLD. *Sci Rep-uk* **11**, 18045 (2021).

6. Gu, Z., Eils, R. & Schlesner, M. Complex heatmaps reveal patterns and correlations in multidimensional genomic data. *Bioinformatics* **32**, 2847–2849 (2016).

7. Yu, G., Wang, L.-G., Han, Y. & He, Q.-Y. clusterProfiler: an R Package for Comparing Biological Themes Among Gene Clusters. *OMICS: A J. Integr. Biol.* **16**, 284–287 (2012).

8. McGinnis, C. S., Murrow, L. M. & Gartner, Z. J. DoubletFinder: Doublet Detection in Single-Cell RNA Sequencing Data Using Artificial Nearest Neighbors. *Cell Syst* **8**, 329-337.e4 (2019).

9. Conde, C. D. *et al.* Cross-tissue immune cell analysis reveals tissue-specific features in humans. *Sci. (N. York, NY)* **376**, eabl5197–eabl5197 (2022).

10. Hao, Y. *et al.* Integrated analysis of multimodal single-cell data. *Cell* **184**, 3573-3587.e29 (2021).

11. Zhao, J. *DAseq: Detecting Regions of Differential Abundance between ScRNA-Seq Datasets*. (2020).

12. Sun, D. *et al.* Identifying phenotype-associated subpopulations by integrating bulk and single-cell sequencing data. *Nat Biotechnol* **40**, 527–538 (2022).

13. Cao, J. *et al.* The single-cell transcriptional landscape of mammalian organogenesis. *Nature* **566**, 496–502 (2019).

14. Korotkevich, G. *et al.* Fast gene set enrichment analysis. *bioRxiv* 060012 (2021) doi:10.1101/060012.
