# Supplementary material for: Single-cell landscape of peripheral immune cells in MASLD/MASH
Source: Hepatol Commun. 2025 Apr 21;9(5):e0643. doi: 10.1097/HC9.0000000000000643 (PMC12014121; doi:10.1097/HC9.0000000000000643)
Supplement: Supplementary file 8 [file hc9-9-e0643-s008.pdf]

SDC, Figure 1

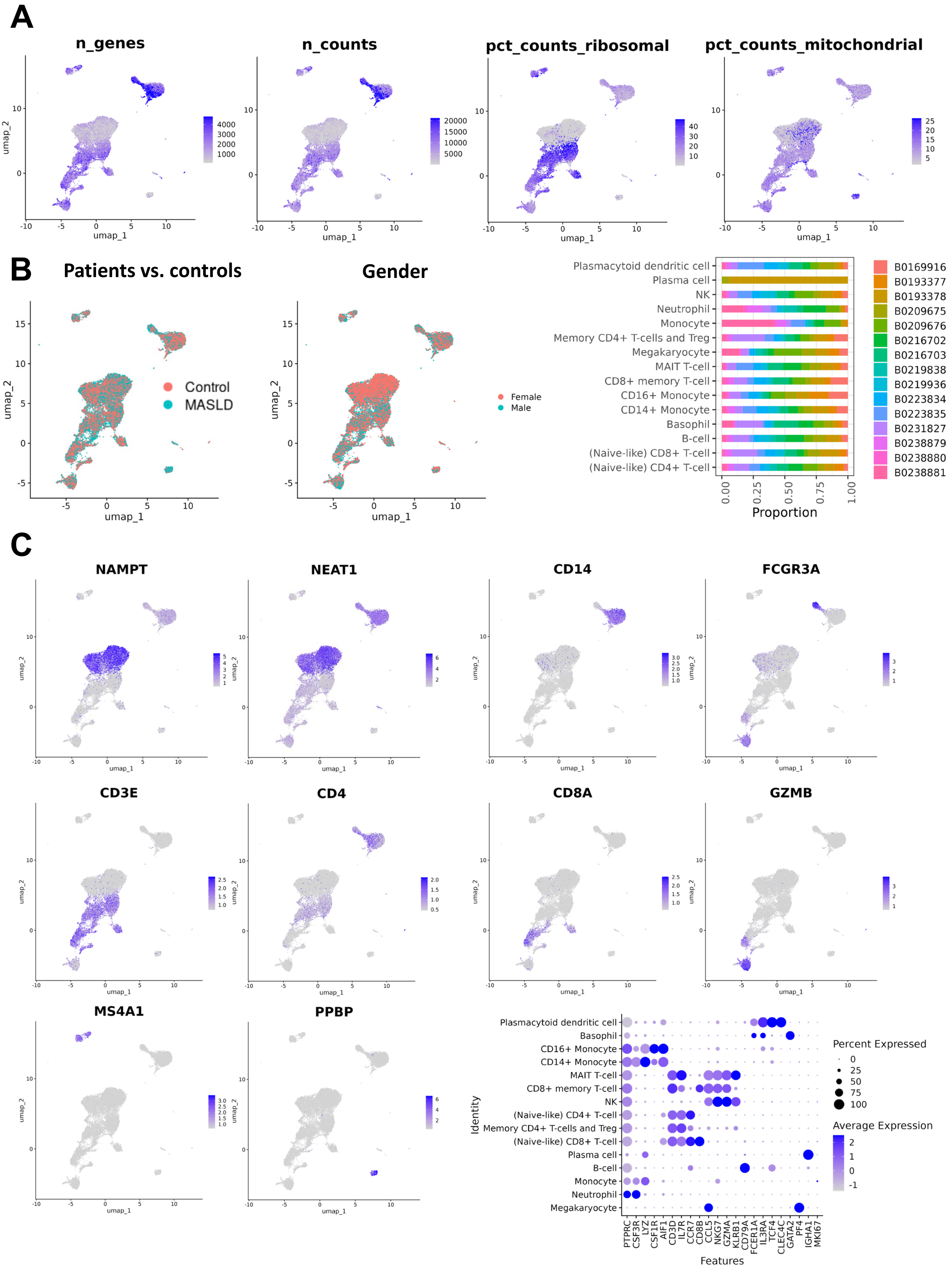

Supp. Fig. 1. (A) UMAP plots showing main quality control features. **(B)** UMAP plots showing integration across health and disease, gender and individual samples. **(C)** Feature plots of markers for major cell populations. **(D)** Dotplot with selected cell type markers.

SDC, Figure 2

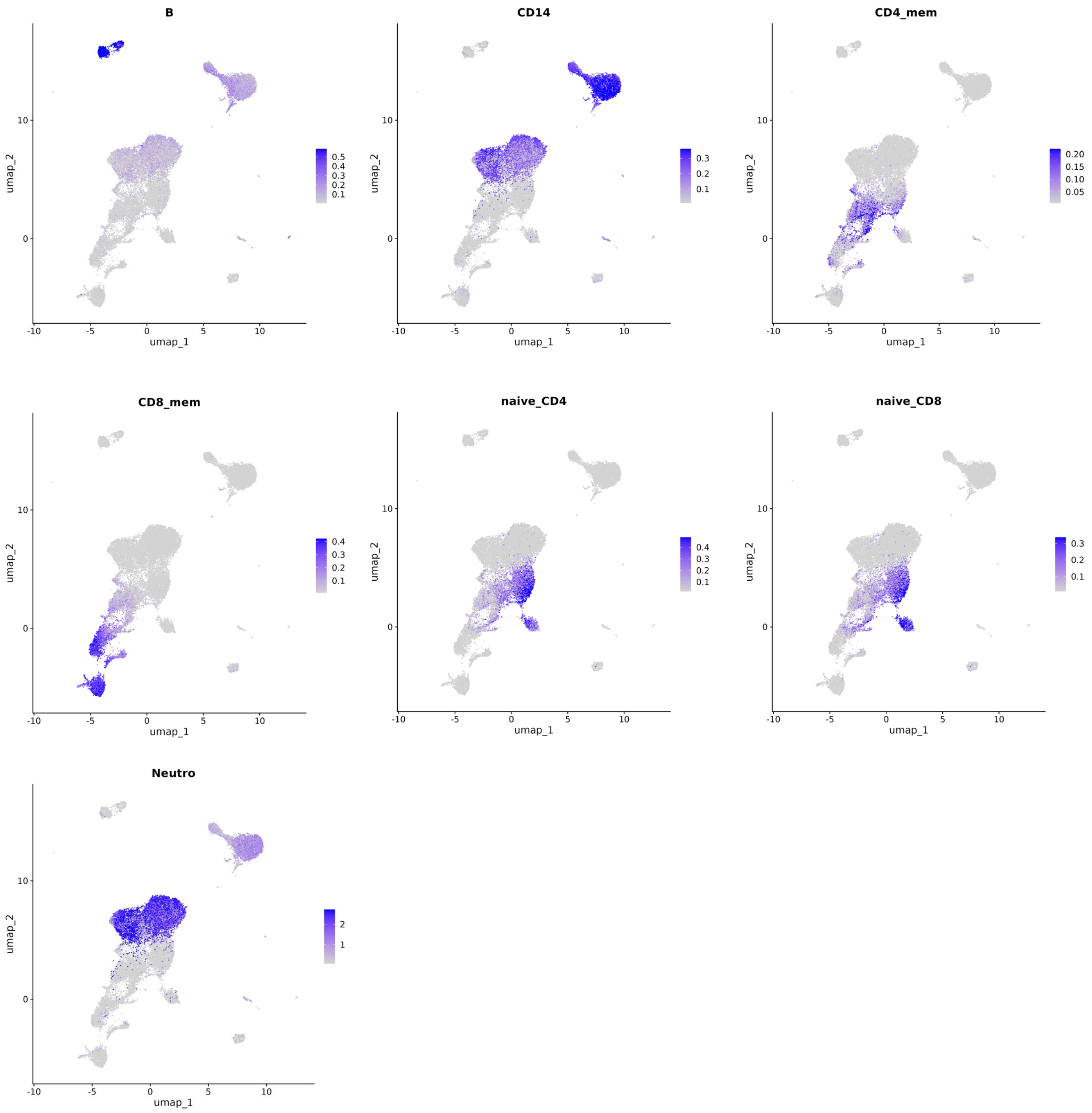

Supp. Fig. 2. Aggregated cell type specificity scores per major cell type population used for enrichment analysis in figure 2B projected onto the UMAP embedding.

SDC, Figure 3

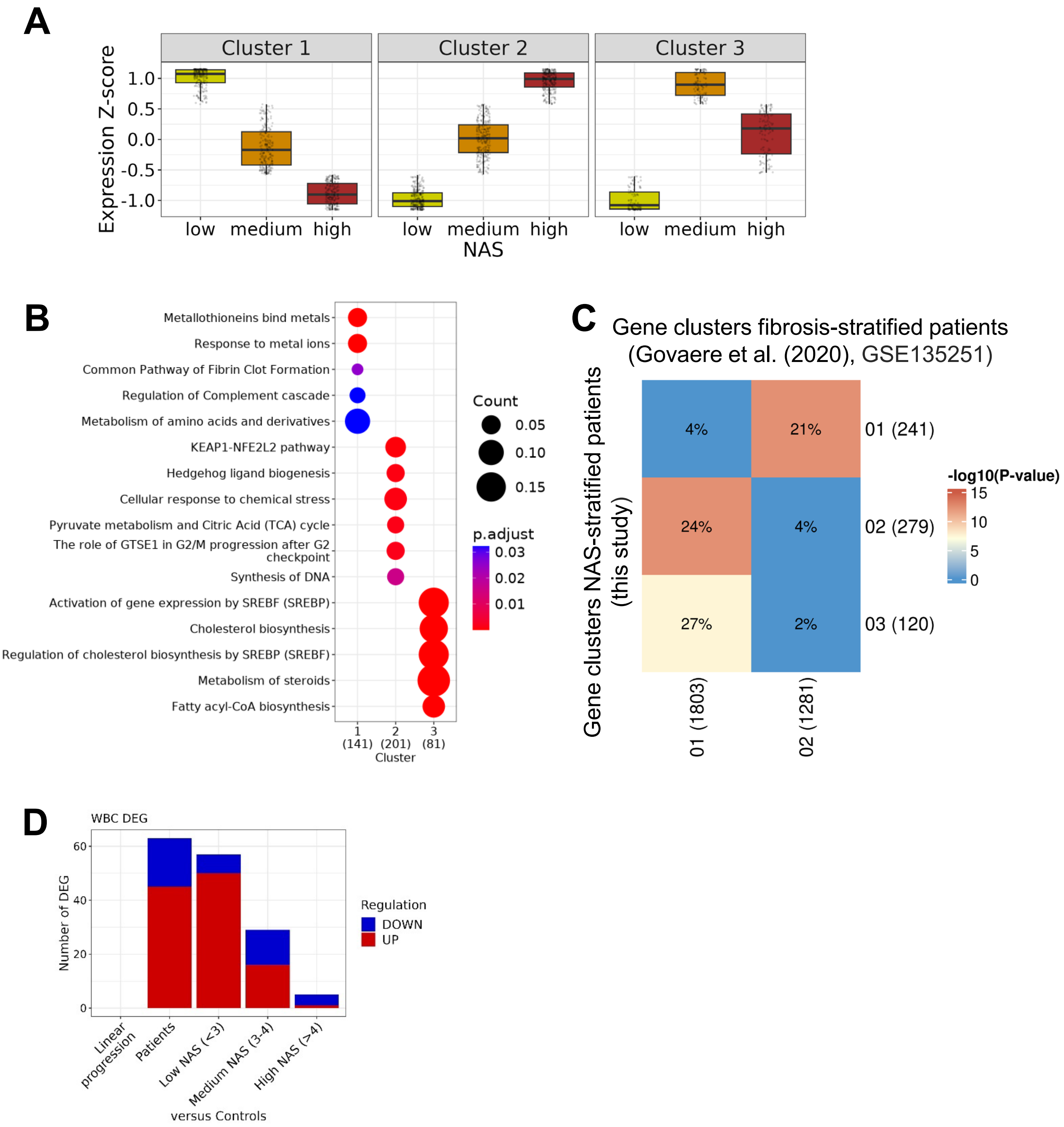

Supp. Fig. 3. Hierarchical cluster analysis of liver bulk DEG and white blood cell DE analysis results. **(A)** Z-score-normalized gene expression of the top three gene clusters identified by hierarchical clustering from liver bulk DE results (pairwise comparison against low NAS). **(B)** Reactome enrichment plot of the top three gene clusters from NAS-stratified patients as shown in (A). Numbers in brackets indicate the number of genes per cluster that could be mapped. **(C)** Pairwise enrichment of gene clusters derived from liver RNAseq from fibrosis-stratified patients (GSE135251) as performed by Sauer et al.[17] and liver RNAseq from NAS-stratified patients. Percentage of overlap between genes in NAS-stratified and fibrosis-stratified clusters shown. Numbers in brackets indicate the number of genes per cluster. **(D)** Number of up/downregulated DEG in white blood cells bulk RNAseq in a linear disease progression model (FDR<0.1, |beta|>1) and two-group comparisons against healthy controls (p_{adj}<0.05, |logFC|>0.25).

SDC, Figure 4

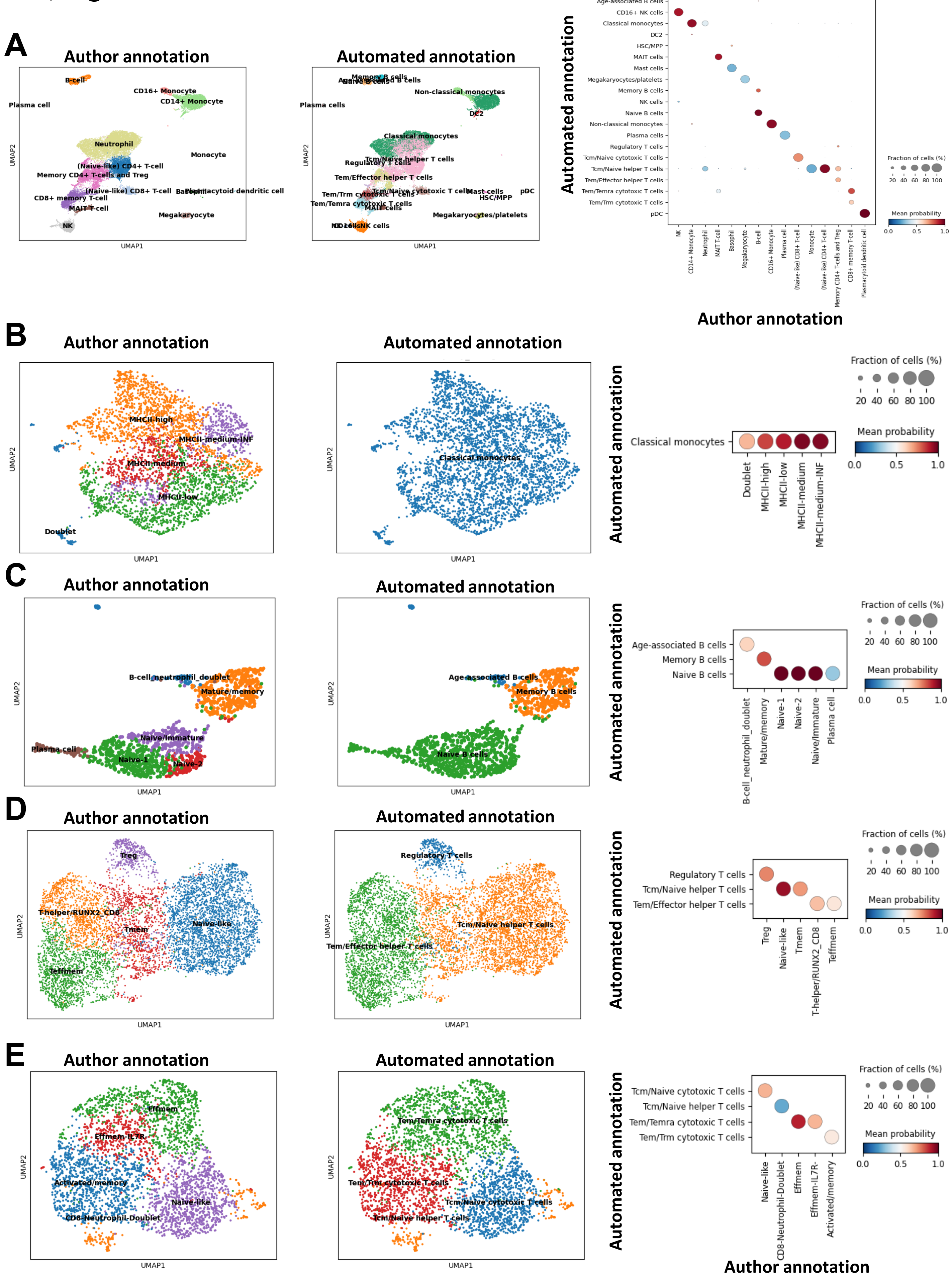

Supp. Fig. 4. Comparison of manual author annotations with automated annotation (CellTypist) for **(A)** the full dataset (note that neutrophils were not in the prediction model), **(B)** CD14+ monocytes, **(C)** B-cells, **(D)** CD4+ T-cells and **(E)** CD8+ T-cells.

SDC, Figure 5

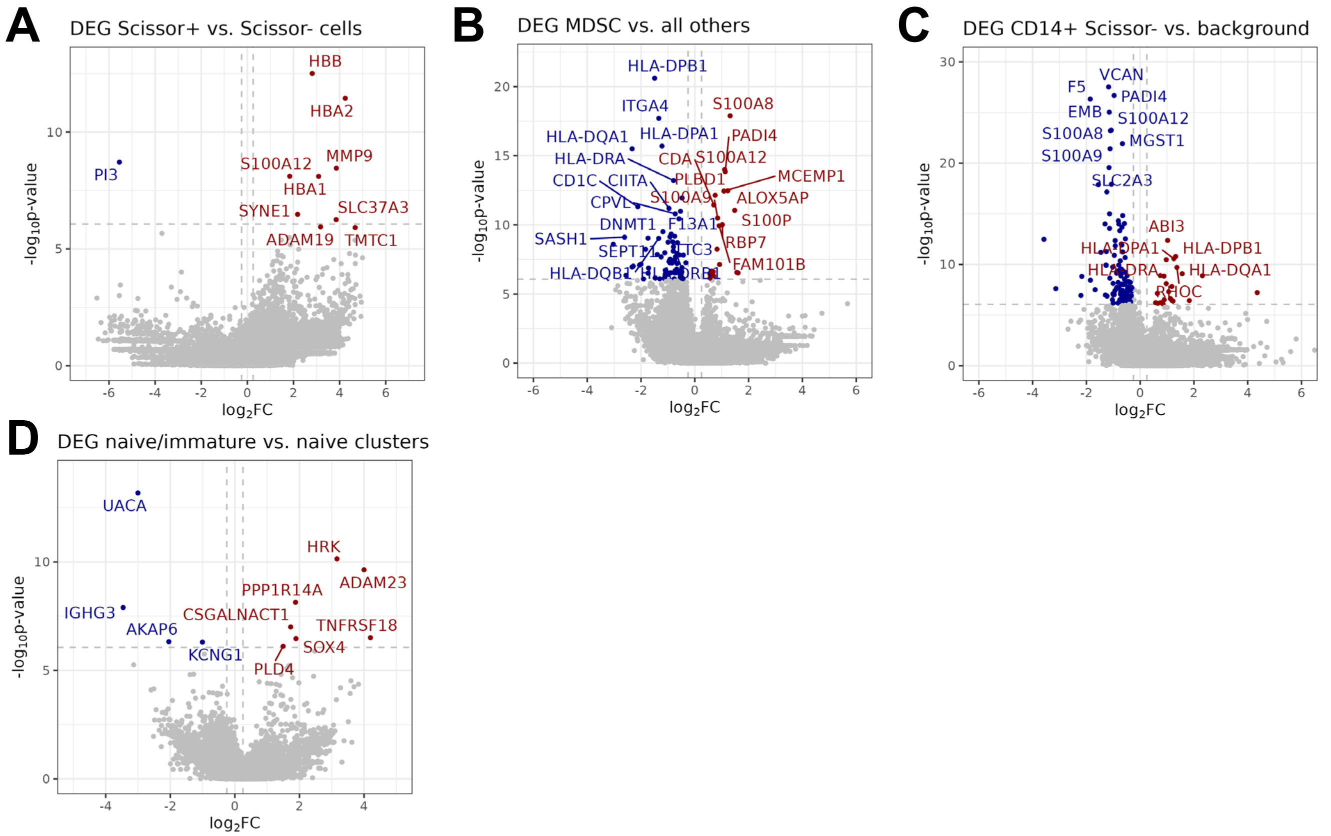

Supp. Fig. 5. Volcano plot of pseudobulk DE testing of **(A)** Scissor+ vs. Scissor- neutrophils, **(B)** MDSC vs. all other CD14+ monocytes, **(C)** Scissor- vs. background CD14+ monocytes and **(D)** naïve/immature vs. naïve B-cell clusters.

SDC, Figure 6

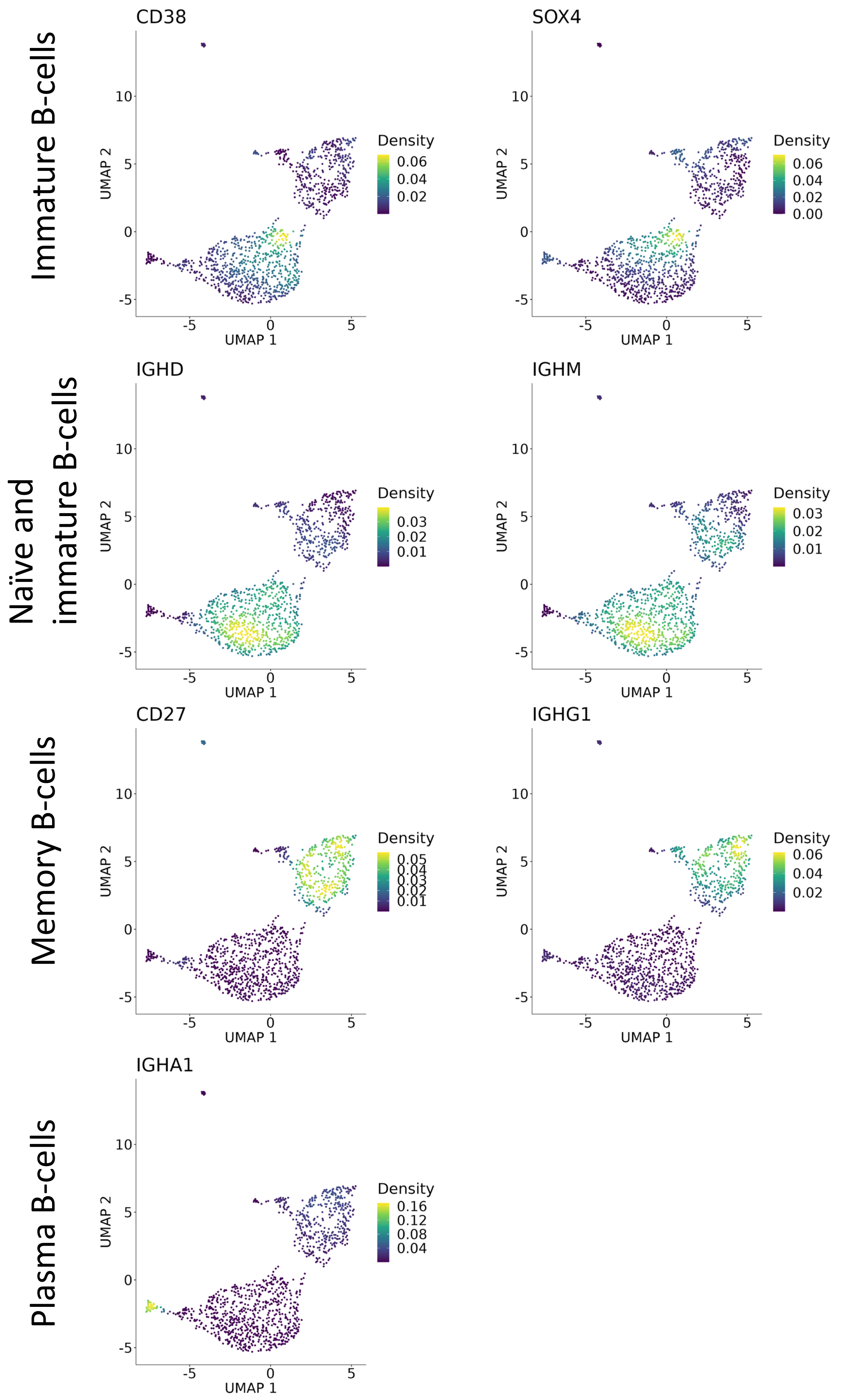

Supp. Fig. 6. Density plots of B-cell subpopulation markers.
